# Mr. Goodwin, on the Prevailing Epidemics

**Published:** 1803-06-01

**Authors:** W. Goodwin

**Affiliations:** Earlsoham, Framlingham, Suffolk


### *To the Editors of the Medical and Physical Journal.*

GENTLEMEN,

A WISH having been expressed in a late Number of the Medical Journal, to know the most prevailing epidemics in the country, I take the first opportunity of sending you the following account of such as have prevailed most generally, in a district of several miles in this part of Suffolk; which, if you think deserving a place in your valuable Miscellany, is at your service.

The most prevailing epidemics for the last twelve months have been typhus maligna and mitior, scarlatina anginosa, measles, and mumps. Many of the former have proved alarmingly fatal in several of our villages, whilst those of the second class of typhoid fevers have put on the appearance of the low nervous kind, attended with great prostration of strength, depression of spirits, loss of appetite, &c. which frequently continue many weeks before a compleat recovery ensues. The most general method of treating these fevers has been by first giving a vitriolic emetic; a small dose of calomel and rhubarb, just sufficient to open the bowels if constipated, after which the cinchona c. serpentaria, aromatics, and red port have been exhibited; but which have generally, in strongly marked cases, not proved sufficiently powerful, without the friendly assistance of the muriatic acid internally, in large quantities, which has succeeded in many desperate cases, beyond all expectation, assisted by the external application of acetum and aqua, keeping the patient constantly wetted therewith by means of a flannel waistcoat and long worsted stockings, applying it either cold or in a tepid state, according to the heat and circumstances attending the patient, invariably premising a frequent change of linen and bedding, ventilating the rooms, and fumigating with vitriolic acid and common salt.

Five cases of the most malignant kind of typhus occurred in a farmer's family I lately attended; the first, in a fine young man of 18, who died after lying several days delirious; mouth, tongue and fauces black; teeth furred, and throat sore. He was treated in the old method, with cortex, cordials, aromatics, and plenty of wine, but without the assistance of acids either internally or externally. About a week after his death, his father, 64 years of age, two grown up sisters, and a brother, fell ill of the same fever; their symptoms, delirium, sore throats, quick, feeble pulse, total loss of appetite and strength, subsultus, black tongues, and full stomachs. They were vomited with zinc. vit. and ipecac.; ordered Port wine, cortex, and aromatics; their rooms ventilated and fumigated; under which treatment they hourly grew from bad to worse; could take very little, and some of them no nourishment, and were given up as lost, particularly the old gentleman, who laid almost perpetually hiccuping in the most violent manner. At this time we commenced our new plan (first suggested to us in your excellent Miscellany) of giving the muriatic acid boldly, which we did by dropping 80 drops of that acid with 20 of elix. vit. and 20 of tinct. opii into a quart of watei, to which was added lemon peel and a sufficient quantity of sugar to make it perfectly grateful and pleasant; of this they drank ad libitum. We likewise prepared another quart, half wine, acidulated as above, of which they also took at pleasure; and for some days little else was taken, as almost every thing but the above disagreed with their stomachs, which were repeatedly so sick and loaded, as to render gentle emetics indispensible.

Two of our patients, for some days, were kept alive by cordial injections per anum, composed of decoct. cinchona, strong broth, wine, and tinct. opii. The old gentleman's hiccup was successfully treated by blistering the diaphragm, and giving two grains of opium, with musk, camphor, and æther at a dose, and repeated as occasion required. All our patients were kept moist with vinegar and warm water, applied from a tea-pot on their flannel waistcoats and long stockings; by the united efforts of which, we had the unexpected pleasure to see them all survive, and by slow degrees recover their former health. One of the nurses out of four, caught the fever and died. Four in a neighbouring family likewise caught it by visiting our patients, two of whom were lost, who being attended by another medical gentleman, had not the acid plan directed. Our four patients took, in about ten days, a pint of muriatic acid and two ounces of elix. of vitriol, making some allowance for the part the nurses might occasionally partake of.

The Scarlet Fever, with and sometimes without the sore throat, has been very rife for several months in a large district around this neighbourhood, and in many parishes has proved very malignant and fatal; some instances having occurred where healthy strong subjects have died in the course of forty-eight hours from the first attack. It is now but little heard of, and 'tis hoped its baneful effects have nearly ceased. The most successful general plan of treating Scarlatina Anginosa has been, to clear the stomach at its commencement with zinc. vit. and ipec.; blistering the throat when sore, gargling with myrrh, infus. cinchonæ and acid, restraining the diarrhœa when profuse by injections of red port, infus. cortex, and t. opii; but above all, by keeping the skin constantly wetted with vinegar and warm water, ventilating and fumigating with vitriolic acid, salt or nitre, changing the bed and clothes when necessary, giving the cortex, serpentaria, and muriatic acid, and allowing a vinous and generous regimen. We have in many forlorn cases of Scarlatina Maligna, as well as Typhus, recovered patients by the free use of acids internally and externally, that were given up as lost, and apparently would have been so, but for their powerful assistance; where they are given largely, a proper quantity of tinct. opii should be subjoined, to prevent their nipping the stomach and bowels.

Several very strong cases of our success in Scarlatina, by the above plan, I could send you, particularly one of a lying-in delicate woman, seized with Scarlatina Anginosa the second day after delivery, with every fatal symptom attending, who was snatched from the jaws of death by the acid plan above described.

I am, &c.

W. GOODWIN.

*Earlsoham, Framlingham, Suffolk, April* 10, 1802.

